# Concerns on cross-species transmission of SARS-CoV-2 between pets and humans

**DOI:** 10.3389/fmicb.2022.985528

**Published:** 2022-09-21

**Authors:** Xingguang Li, Haizhou Liu, Yigang Tong

**Affiliations:** ^1^Hwa Mei Hospital, University of Chinese Academy of Sciences, Ningbo, China; ^2^Ningbo Institute of Life and Health Industry, University of Chinese Academy of Sciences, Ningbo, China; ^3^National Virus Resource Center, Wuhan Institute of Virology, Chinese Academy of Sciences, Wuhan, China; ^4^College of Life Science and Technology, Beijing University of Chemical Technology, Beijing, China

**Keywords:** SARS-CoV-2, COVID-19, cross-species transmission, pets, humans

Coronavirus disease 2019 (COVID-19) is a respiratory illness caused by severe acute respiratory syndrome coronavirus 2 (SARS-CoV-2). The disease and virus were first reported in December 2019 in Wuhan, Hubei province, China, with a serious subsequent impact on global public health (Zhu et al., 2020). As of 23 August 2022, the World Health Organization (WHO) has reported a total of 594,367,247 confirmed cases of COVID-19 globally, including 6,451,016 deaths (https://www.who.int/emergencies/diseases/novel-coronavirus-2019) and more than 12 billion doses of vaccines. At least 30 new infectious agents impacting humans have emerged over the past 35 years, including Middle East respiratory syndrome coronavirus (MERS-CoV), severe acute respiratory syndrome coronavirus (SARS-CoV), and SARS-CoV-2. Notably, 61% of infectious organisms affecting humans are zoonotic in nature, i.e., can infect both humans and animals (Nii-Trebi, 2017; McArthur, 2019).

As of 21 August 2022, a total of 2,071 SARS-CoV-2 sequences sampled from 23 animals (mink, *n* = 1 318; deer, *n* = 318; cat, *n* = 131; dog, *n* = 90; lion, *n* = 71; tiger, *n* = 43; mouse, *n* = 23; hamster, *n* = 17; gorilla, *n* = 15; snow leopard, *n* = 11, bat, *n* = 9; otter, *n* = 8; monkey, *n* = 4; ferret, *n* = 3; swan, *n* = 2; binturong, *n* = 1; coati, *n* = 1; fishing cat, *n* = 1; hippopotamus, *n* = 1; hyena, *n* = 1; leopard, *n* = 1; pangolin, *n* = 1; puma, *n* = 1) in 39 countries (United States, *n* = 794; Denmark, *n* = 471; the Netherlands, *n* = 293; Latvia, *n* = 198; Lithuania, *n* = 46; Spain, *n* = 34; Poland, *n* = 33; China, *n* = 30; Switzerland, *n* = 30; France, *n* = 20; Austria, *n* = 18; Brazil, *n* = 17; Canada, *n* = 13; India, *n* = 8; Thailand, *n* = 5; Laos, *n* = 5; Germany, *n* = 5; Czech Republic, *n* = 5; Russia, *n* = 4; Italy, *n* = 4; Colombia, *n* = 4; Argentina, *n* = 4; Sweden, *n* = 3; South Africa, *n* = 3; Peru, *n* = 3; Bosnia and Herzegovina, *n* = 3; Belgium, *n* = 3; Belarus, *n* = 3; Puerto Rico, *n* = 2; United Kingdom, *n* = 1; Sri Lanka, *n* = 1; Slovenia, *n* = 1; Singapore, *n* = 1; Portugal, *n* = 1; Mexico, *n* = 1; Iran, *n* = 1; Greece, *n* = 1; Egypt, *n* = 1; Botswana, *n* = 1) and belonging to various lineages have been submitted to the GISAID platform (Elbe and Buckland-Merrett, 2017) (https://public.tableau.com/app/profile/raj.rajnarayanan/viz/COVID19DASHBOARDANIMALSGLOBAL/AnimalCov-Global; Figure 1 and Supplementary Table S1). The most sequenced specimens have been sampled from mink and deer (1,636/2,070; 79.03%) in the United States and Denmark (1,264/, 070; 61.06%), respectively. At present, in the United States, a total of 387 SARS-CoV-2 infections have been confirmed in pets and other animals under human care (cat, *n* = 118; dog, *n* = 111; lion, *n* = 53; tiger, *n* = 53; gorilla, *n* = 21; snow leopard, *n* = 13; otter, *n* = 8; spotted hyena, *n* = 2) (https://www.aphis.usda.gov/aphis/dashboards/tableau/sars-dashboard).

**Figure 1:**
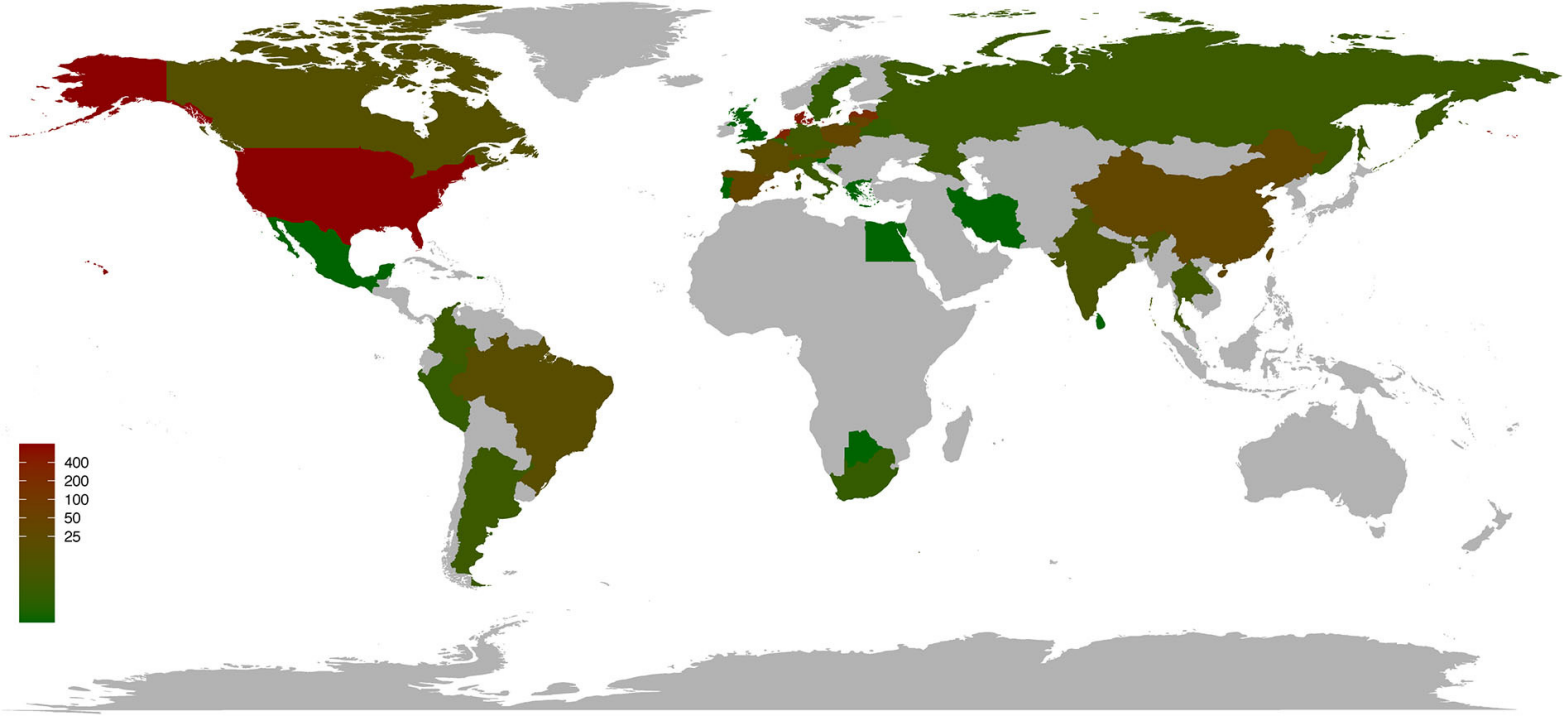
Worldwide distribution of SARS-CoV-2 infections in animals by country. As of 21 August 2022, 39 countries have reported confirmed cases of SARS-CoV-2 in animals. Data were obtained from COVID19 DASHBOARD ANIMALS GLOBAL (https://public.tableau.com/app/profile/raj.rajnarayanan/viz/COVID19DASHBOARDANIMALSGLOBAL/AnimalCov-Global).

Human-to-pet transmission of SARS-CoV-2 has been reported in many countries (https://www.cdc.gov/coronavirus/2019-ncov/daily-life-coping/animals.html, https://www.woah.org/en/what-we-offer/emergency-and-resilience/covid-19/#ui-id-3), especially in domestic cats (Shi et al., 2020; Zhang et al., 2020) and dogs (Sit et al., 2020; Barroso et al., 2022). Furthermore, cats show efficient viral replication and can transmit SARS-CoV-2 to each other *via* respiratory droplets (Shi et al., 2020). Research has also shown that 14.7% of recently collected cat serum samples (15 out of 102) are positive for the receptor-binding domain (RBD) of SARS-CoV-2 (Zhang et al., 2020), indicating that the virus may be pervasive in cats living closely with infected people. Dogs have also tested positive for SARS-CoV-2 following infection from humans (Sit et al., 2020; Barroso et al., 2022), further suggesting that people infected with SARS-CoV-2 should not only avoid contact with other people but also with wildlife, livestock, and pets. At present, 143 and 96 sequences of SARS-CoV-2 sampled from cats and dogs belonging to at least 60 and 40 lineages, respectively, have been submitted to the GISAID platform (Elbe and Buckland-Merrett, 2017) (Supplementary Table S2). Based on current data, human-to-pet transmission is more common than pet-to-human transmission. However, cross-species transmission from pets to humans has been reported, e.g., hamsters in Hong Kong (Yen et al., 2022) and cats in Thailand (Sila et al., 2022). Although SARS-CoV-2 infection in pets remains poorly understood, COVID-19 outbreaks will be inevitable and difficult to eradicate if animals become permanent reservoirs. Previous research suggests that the Omicron variant may have evolved in a mouse host (Sun et al., 2022). Notably, Omicron can bind to mouse angiotensin-converting enzyme 2 (ACE2) better than previous VOCs (Peacock et al., 2022), indicating the possibility of species differences in infectivity, which is a concern that needs to be addressed. As such, understanding which animals can be infected with SARS-CoV-2 and their potential role in cross-species transmission is critical. Furthermore, as abandoned infected pets are likely to become an issue for cities, several provincial regulations on animal epidemic prevention have been introduced, e.g., regulations on dog breeding enacted on 1 March 2022 in Hubei province, China (http://www.chinadaily.com.cn/a/202203/07/WS62257859a310cdd39bc8adc3.html). According to the revisions, people who abandon dogs or cats may face fines of 1,000–5,000 RMB. Residents are also prohibited from raising “large and dangerous” dogs, with fines of up to 10,000 RMB and removal of the pet. Such revisions are likely to be enacted in other provinces of China as well as for other pets.

Although confirmed cases of COVID-19 in humans have exceeded 594 million (https://www.who.int/emergencies/diseases/novel-coronavirus-2019), the real number of infected people worldwide may be as much as 10 times higher (https://medicalxpress.com/news/2020-04-covid-average-actual-infections-worldwide.html). Notably, asymptomatic carriers are not uncommon, and the nucleic acid of SARS-CoV-2 in convalescent patients can re-test positive, suggesting viral preservation. Such factors may contribute to subsequent COVID-19 outbreaks. Whether pets play a role in the transmission and their potential fate following infection remains unclear, especially given the history of animal euthanasia for disease control (Galvin et al., 2005; Raj, 2008; Thornber et al., 2014). Therefore, clarifying the range of pet species that can be infected by and transmit to humans is important. At present, there is no available vaccine to protect pets from SARS-CoV-2 (https://www.cdc.gov/coronavirus/2019-ncov/downloads/covid-19-pets-prevention.pdf). Thus, we recommend increasing public awareness on the responsibilities of pet ownership and feeding stray animals (i.e., following all safety and social distancing guidelines). Certainly, better pet care should be promoted rather than abandoning or killing of pets with SARS-CoV-2 (https://www.nature.com/articles/d41586-022-01792-y, https://www.cdc.gov/healthypets/covid-19/pets.html#guidance, https://www.cdc.gov/coronavirus/2019-ncov/downloads/covid-19-pets-prevention.pdf). In summary, better legislation regulating pet ownership and abandonment is required to strengthen the monitoring, isolation, and treatment of infected pets, as well as potential pet-to-human and human-to-pet transmissions, and to prevent the formation of animal reservoirs. Increasing public awareness on proper hygiene practices regarding close contact with pets, other animals, their droppings, and human waste is essential to reduce the risk of cross-species transmission of SARS-CoV-2 (https://www.who.int/news/item/07-03-2022-joint-statement-on-the-prioritization-of-monitoring-SARS-CoV-2-infection-in-wildlife-and-preventing-the-formation-of-animal-reservoirs). Our attitudes to pets and their care are important not only for reducing the spread of COVID-19 but also for reducing the potential evolution of novel variants.

## Author contributions

XL conceived and designed the study and drafted the manuscript. XL, HL, and YT reviewed and approved the final version of the manuscript. All authors contributed to the article and approved the submitted version.

## Funding

This study was supported by the National Key Research and Development Project of China (No. 2021YFC0863400) granted to YT.

## Conflict of interest

The authors declare that the research was conducted in the absence of any commercial or financial relationships that could be construed as a potential conflict of interest.

## Publisher's note

All claims expressed in this article are solely those of the authors and do not necessarily represent those of their affiliated organizations, or those of the publisher, the editors and the reviewers. Any product that may be evaluated in this article, or claim that may be made by its manufacturer, is not guaranteed or endorsed by the publisher.
